# Advances in IVUS/OCT and Future Clinical Perspective of Novel Hybrid Catheter System in Coronary Imaging

**DOI:** 10.3389/fcvm.2020.00119

**Published:** 2020-07-31

**Authors:** Masafumi Ono, Hideyuki Kawashima, Hironori Hara, Chao Gao, Rutao Wang, Norihiro Kogame, Kuniaki Takahashi, Ply Chichareon, Rodrigo Modolo, Mariusz Tomaniak, Joanna J. Wykrzykowska, Jan J. Piek, Isao Mori, Brian K. Courtney, William Wijns, Faisal Sharif, Christos Bourantas, Yoshinobu Onuma, Patrick W. Serruys

**Affiliations:** ^1^Department of Clinical and Experimental Cardiology, Heart Center, Amsterdam Cardiovascular Sciences, Amsterdam UMC, University of Amsterdam, Amsterdam, Netherlands; ^2^Department of Cardiology, National University of Ireland, Galway (NUIG), Galway, Ireland; ^3^Department of Cardiology, Radboud University, Nijmegen, Netherlands; ^4^Depatrment of Cardiology, Xijing hospital, Xi'an, China; ^5^Division of Cardiology, Department of Internal Medicine, Faculty of Medicine, Prince of Songkla University, Songkhla, Thailand; ^6^Cardiology Division, Department of Internal Medicine, University of Campinas (UNICAMP), Campinas, Brazil; ^7^Thoraxcentre, Erasmus Medical Centre, Rotterdam, Netherlands; ^8^First Department of Cardiology, Medical University of Warsaw, Warsaw, Poland; ^9^Terumo Corporation, Tokyo, Japan; ^10^Schulich Heart Program, Sunnybrook Research Institute, University of Toronto, Toronto, ON, Canada; ^11^Conavi Medical, North York, ON, Canada; ^12^Barts Heart Centre, Barts Health NHS Trust, London, United Kingdom

**Keywords:** intravascular ultrasound, optical coherence tomography, hybrid IVUS–OCT catheter, intracoronary imaging, vulnerable plaque, percutaneous coronary intervention

## Abstract

Intravascular ultrasound (IVUS) and optical coherence tomography (OCT) have been developed and improved as both diagnostic and guidance tools for interventional procedures over the past three decades. IVUS has a resolution of 100 μm with a high tissue penetration and capability of assessing the entire structure of a coronary artery including the external elastic membrane, whereas OCT has a higher resolution of 10–20 μm to assess endoluminal structures with a limited tissue penetration compared to IVUS. Recently, two companies, CONAVI and TERUMO, integrated IVUS and OCT into a single catheter system. With their inherent strength and limitations, the combined IVUS and OCT probes are complementary and work synergistically to enable a comprehensive depiction of coronary artery. In this review, we summarize the performance of the two intracoronary imaging modalities—IVUS and OCT—and discuss the expected potential of the novel hybrid IVUS–OCT catheter system in the clinical field.

## Introduction

### History of Intracoronary Imaging Modalities

**Figure 1:**
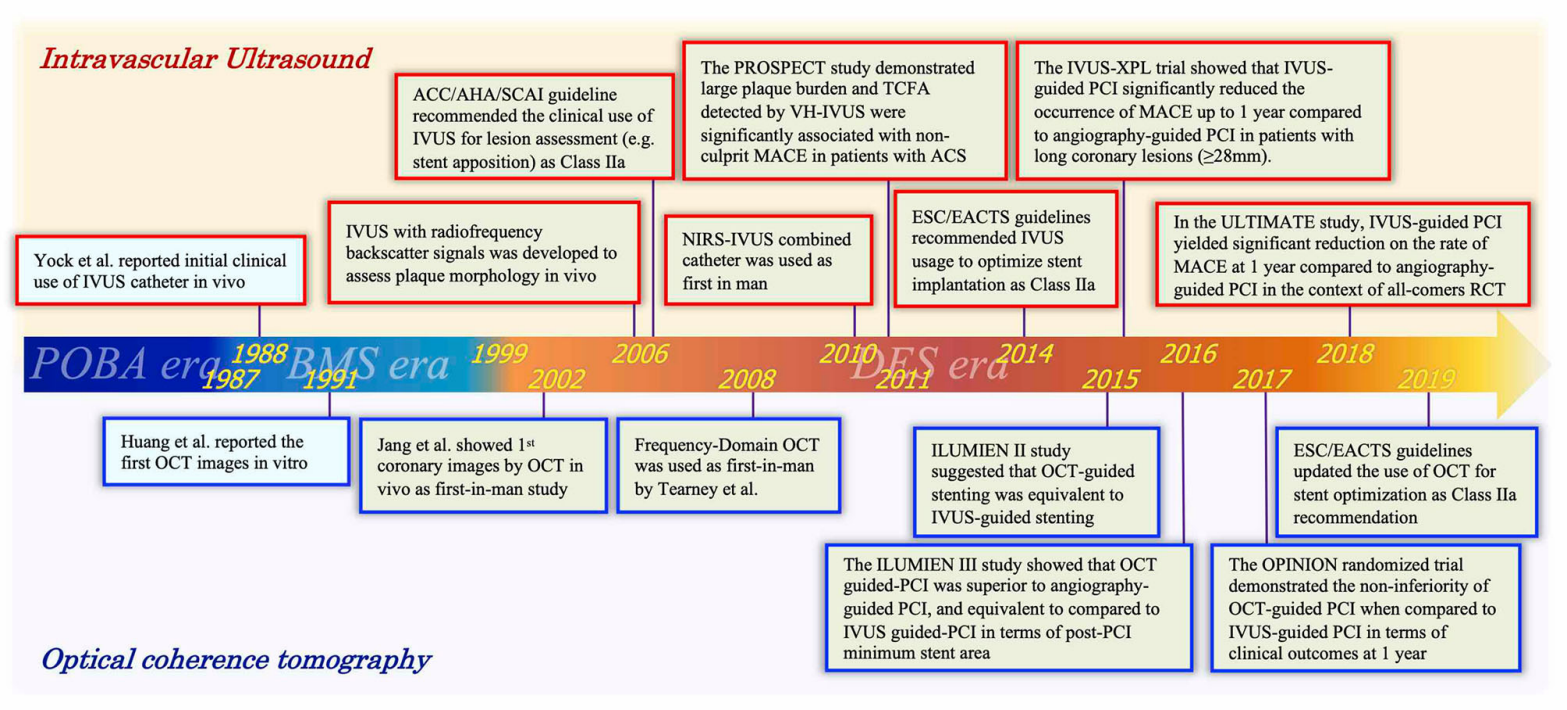
Timeline of intravascular ultrasound and optical coherence tomography with advances of PCI era. A history focused on intracoronary imaging devices (IVUS and OCT). Red and blue frames indicate representative events associated with IVUS and OCT, respectively. IVUS, intravascular ultrasound; OCT, optical coherence tomography; POBA, plain old balloon angioplasty; BMS, bare-metal stent; DES, drug-eluting stent; PCI, percutaneous coronary intervention; ACC, American College of Cardiology; AHA, American Heart Association; SCAI, Society for Cardiovascular Angiography; ESC, European Society of Cardiology; EACTS, European Association for Cardio-Thoracic Surgery; MACE, major adverse cardiac events.

The history of percutaneous coronary intervention (PCI) started with the first coronary balloon angioplasty performed by Andreas Grüntzig in 1977 (1). In parallel with the remarkable evolution of PCI and the development of drug-eluting stents (DESs), the performance of intravascular imaging devices has been also improved. Figure 1 summarizes the history of both interventional cardiology and intravascular imaging devices. Intravascular ultrasound (IVUS), as the first intravascular imaging device, was introduced by Yock et al. in the 1980s (2). Optical coherence tomography (OCT) was introduced a few years later in the 1990s (3, 4). Although the two devices have the same basic principles and visualize the intracoronary structures by reconstructing images from signal waves scattered back from the vessel wall to the catheter, the utilized signals are different: ultrasound (wavelength 40–50 μm) in IVUS and low-coherence light (wavelength 1.3 μm) in OCT (5, 6). The two modalities of imaging have advantages and disadvantages, which are described in the next chapter (1-2).

In 1999, the first DES was introduced that lowered the rate of in-stent restenosis compared to bare-metal stents (BMSs) (7–11). In the new era of DESs, not only simple lesions but also more complex lesions were treated by PCI; in this setting, intravascular imaging has played an important role as a clinical support tool for planning and assessing the final results of PCI (12, 13). In the IVUS-XPL trial, Hong et al. demonstrated that usage of IVUS significantly reduced 1- and 5-year major adverse cardiac events (MACE; cardiac death, target vessel myocardial infarction [MI], or ischemic-driven target lesion revascularization) in patients with long lesions implanted with second-generation DES, which proved the efficacy and safety of the usage of IVUS in complex PCI (14, 15). Moreover, Zhang et al. reported that IVUS-guided DES implantation significantly reduced target-vessel failure (TVF: cardiac death, target-vessel MI, or ischemic-driven target vessel revascularization) at 12 months compared to angiography-guided DES implantation in the ULTIMATE (Intravascular Ultrasound Guided Drug Eluting Stents Implantation in “All- Comers” Coronary Lesions) randomized controlled trial (16). Those results support the use of IVUS as a PCI guidance tool in the contemporary PCI era.

In addition to conventional gray-scale IVUS assessment, analysis of the raw backscattered IVUS radiofrequency (RF) data can be also utilized for assessments of plaque morphology, tissue characterization, and vulnerable plaque detection (17). In 2011, Stone et al. reported the results of the PROSPECT (Providing Regional Observations to Study Predictors of Events in the Coronary Tree) study, demonstrating that a large plaque burden (≥70%) (HR: 5.03, 95% CI: 2.51–10.11) and a thin-cap fibroatheroma (TCFA) (HR: 3.35, 95% CI: 1.77–6.36) detected by IVUS-RF as well as a minimal lumen area <4.0 mm^2^ (HR: 3.21, 95% CI: 1.61–6.42) were independent predictors of MACE (cardiac death, cardiac arrest, MI, or rehospitalization due to unstable or progressive angina) in non-culprit lesions of patients presented with acute coronary syndrome (ACS) (18). In this trial, the outcome of MACE was mainly driven by the incidence of rehospitalization due to unstable or progressive angina. Thereafter, the ATHEROREMO-IVUS (European Collaborative Project on Inflammation and Vascular Wall Remodeling in Atherosclerosis—Intravascular Ultrasound) study confirmed that the finding of VH-IVUS TCFA was independently associated with the composite of death and ACS (19). Those trials unraveled the importance of assessing plaque characteristics in a high-risk population, and the efficacy of IVUS-RF on stratifying cardiovascular risk, apart from clinical and angiographic characteristics (20).

The first-in-man OCT study was conducted in 2002 (21). In the ILUMIEN II study, OCT-guided stent implantation showed comparable stent expansions (defined as the minimal stent area divided by the mean of the proximal and distal reference lumen areas) compared to those reported in the IVUS-guided group of the ADAPT-DES study (72.8 vs. 70.6%) (22). Thereafter, the ILUMIEN III: OPTIMIZE PCI randomized study showed a comparative efficacy of OCT-guided PCI with a reference of IVUS-guided PCI in terms of a post-PCI minimum stent area (non-inferiority margin: 1.0 mm^2^, one-sided 97.5% lower CI: 0.70 mm^2^, p for non-inferiority = 0.001, p for superiority = 0.42) (23). With regard to clinical outcomes, the OPtical frequency domain imaging vs. INtravascular ultrasound in percutaneous coronary InterventiON (OPINION) randomized controlled trial showed that OCT-guided PCI was not inferior to the IVUS-guided PCI in terms of TVF (cardiac death, target-vessel MI, or ischemia-driven target vessel revascularization) at 12 months follow-up (HR: 1.07, upper limit of one-sided 95% CI: 1.80, p for non-inferiority = 0.042) (24). The latest European Society of Cardiology (ESC)/EACTS guidelines updated the indication of usage of OCT for stent optimization as class IIa recommendation, corresponding with the same level of recommendation as IVUS (25).

Recently, the CLIMA study demonstrated that the OCT-defined plaque vulnerability features (MLA <3.5 mm^2^, TCFA, lipid arc circumferential extension >180°, and macrophage findings) in the left anterior descending artery (LAD) were significantly associated with the increased risk of a composite of cardiac death and target-segment (LAD) MI at 12 months among patients undergoing clinically indicated coronary angiography (HR: 7.54, 95% CI: 3.1–18.6) (26). The results of the CLIMA study indicated the feasibility and efficacy of OCT for detecting high-risk plaques leading to adverse events. Further trials such as the ILUMIEN IV: Optimal PCI trial (NCT03507777) and October trial (NCT03171311) (27) are expected to further demonstrate the clinical safety and efficacy of OCT in guiding PCI.

IVUS and OCT have evolved in parallel over the last years. Recently, however, hybrid IVUS-OCT systems were developed to merge the advantages of both modalities into a single catheter (28–30).

This review aims to summarize the differences and complementary aspects of IVUS and OCT, and describe the novel hybrid IVUS–OCT catheter systems.

### Basic Advantages and Disadvantages of IVUS and OCT

Although both IVUS and OCT have similarities, it is still controversial which intravascular imaging modality is better to be used as either a diagnostic or guidance tool (6, 31). Multimodality invasive imaging assessments are not recommended due to the increased risk of complications and cost. Therefore, operators should be aware of each modality's advantages and disadvantages, and select the imaging tool based on the clinical need, location of the segment of interest, angiographic appearance of the vessel, lesion characteristics, and operators' experiences. In fact, a recent web-based survey suggested that most operators thought to use each imaging modality (IVUS and OCT) properly depending on the specific anatomic and patient characteristics (32).

#### Imaging Acquisition

One of the disadvantages of OCT is the need for blood clearance during pull-back since the light signal is attenuated by the red blood cells (6). Blood clearance is achieved by contrast injection. The total volume of the contrast medium tends to be higher in OCT-guided PCI than those without OCT. In the ILUMIEN III: OPTIMIZE PCI study, the contrast volume during the PCI was significantly higher in the OCT group compared to that of the IVUS group (median 222 vs. 190 ml, *p* = 0.004) or the angiography group (vs. 183 ml, *p* = 0.001) (23). Therefore, in patients with renal impairment, IVUS should be preferred over OCT (23, 24). Studies suggested alternatives to contrast agents for blood clearance during OCT pull-back (33), such as low-molecular-weight dextran (LMWD) to perform “zero-contrast PCI” (34–37). On the other hand, IVUS does not require blood clearance, and it has been shown that it can minimize the contrast volume when compared to angiography-guided PCI (38). In the ULTIMATE trial, however, IVUS-guided PCI was associated with significantly higher contrast volume than angiography-guided PCI (16). Since this paradox was possibly due to longer procedural time for stent optimization in the IVUS-guided PCI group, operators should pay attention to the use of contrast during IVUS-guided PCI as well.

#### Resolution and Penetration Depth

**Figure 2:**
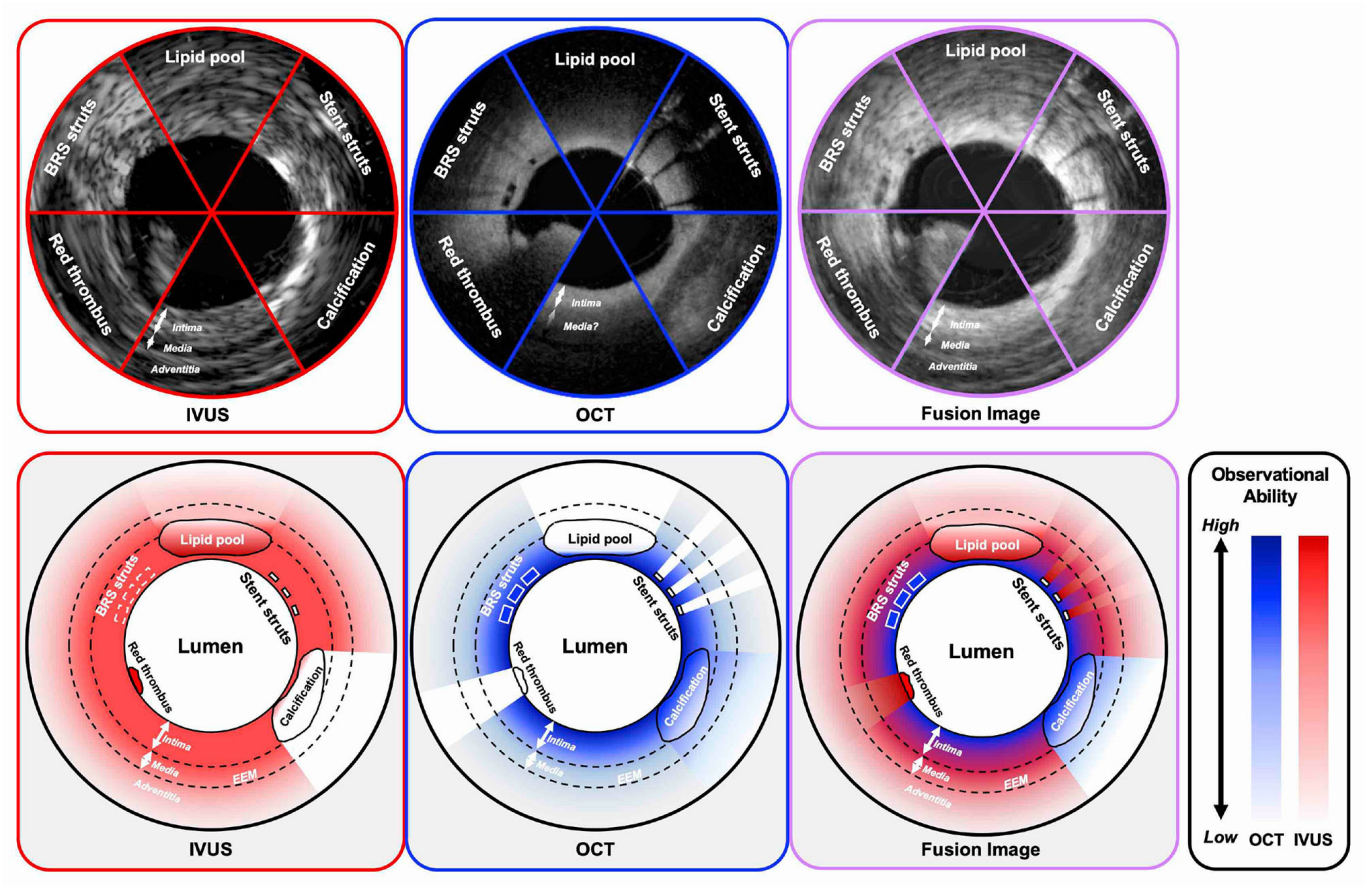
Observational abilities of IVUS and OCT in the settings of several intracoronary structures. IVUS indicates both grayscale IVUS and IVUS with radiofrequency analysis. IVUS, intravascular ultrasound; OCT, optical coherence tomography; BRS, bioresorbable scaffold; EEM, external elastic membrane.

With respect to the imaging capabilities of intracoronary imaging, resolution, and penetration depth are recognized as two important factors (Figure 2). OCT has the highest resolution among all the contemporary available coronary imaging modalities (axial 10–20 μm and lateral 20–90 μm), which is ~10 times greater than that of IVUS (axial 100–150 μm and lateral 150–300 μm) (4, 39). The higher resolution of OCT enables more detailed evaluation than IVUS at the endoluminal level and for the superficial plaque (e.g., in detecting cavity formation of a plaque rupture, TCFA, and stent architecture) (40). With regards to its diagnostic performance of functionally significant stenosis, Ramasamy et al. reported that OCT has a better accuracy than IVUS in non-left main stem lesions from the meta-analysis of 33 studies (41). Moreover, the high resolution of OCT enables the identification of suboptimal PCI results such as coronary dissections, tissue protrusions, underexpansion, and stent struts malapposition more clearly than IVUS (23). Furthermore, also in the long-term follow-up, OCT can evaluate the neointimal proliferation, neoatherosclerosis, uncovered struts, persistent/late-acquired stent malapposition, and/or coronary evagination, which would be associated with adverse events (42–44). In the consensus paper from the European Association of Percutaneous Cardiovascular Interventions (EAPCI) published in 2018, the use of OCT was highly recommended in the case of stent failures since most of the causes of those stent failures could be detected by OCT (45, 46).

In contrast to IVUS, OCT has lower tissue penetration depth (1–2 mm) than IVUS (5–6 mm), leading to incomplete visualization of the vessel wall especially in large vessels, or in case of an increased plaque burden (47). In this context, IVUS enables assessment of the deeper layers of the vessel than OCT, including adventitia. Proper vessel and stent sizing are important parts for PCI optimization because the under- or overestimation of the stent size can lead to suboptimal results or complications such as major coronary dissection, perforation or extensive malapposition, and underexpansion (42, 48, 49). In IVUS, the external elastic membrane (EEM)-based approach for sizing is often used, in which the smallest EEM diameter in the reference area is recommended as the stent reference diameter. The approach may not be always feasible in OCT due to its limited penetration depth. In the ILUMEN III study, the distal EEM was visible for >180° in 76.8% cases by site-assessed and in 95.2% cases by core lab-assessed (23). The measurement protocol of OCT for reference stent diameters is expected to be refined in further clinical studies. By using IVUS, we can assess all layers of interest of the vessel including vessel remodeling in the follow-up imaging (50, 51). Radiofrequency assessment or echogenicity may potentially help in differentiating the components of plaques (52).

#### Calcification

Ultrasound is significantly influenced by the presence of calcium; calcified plaque scatters ultrasound signal, and therefore, the evaluation of plaque behind calcium is not feasible by IVUS (53). On the other hand, with OCT, the calcified plaques are recognized as low-intensity structures with clear demarcation of the calcific tissue borders (54). In the context of clinical implication, Maejima et al. reported that a calcium arc below 227 degrees and a calcium thickness below 670 μm would suggest the use of cutting/scoring balloons (55). Recently, an OCT-based calcium scoring system was developed to predict stent underexpansion (56). The calcium score is composed of maximum angle of >180° (2 points), maximum thickness of >0.5 mm (1 point), and length of >5 mm (1 point). The calcium score of 4 was significantly associated with stent underexpansion when compared to a score of 0–3 (78 vs. 96%, *p* < 0.01), suggesting heavily calcified lesions that need debulking.

#### Three-Dimensional Reconstruction

Online three-dimensional (3D) reconstruction is one optional advantage of OCT. It can assist in understanding the complex structures in some specific cases (e.g., a stent fracture), which is difficult to be detected in cross-sectional two-dimensional images (57–60). Although a 3D reconstruction image is also possible in IVUS, the tedious segmentation process and its poor resolution that does not allow sufficient strut-level assessment render IVUS an unattractive modality of 3D vessel modeling (61, 62).

#### Physiological Assessment

Recently, physiological assessment of lesion severity was attempted by processing IVUS and OCT data using computational modeling (63–67). The accuracy of these approaches strongly depends on the accuracy of the 3D imaging models. Therefore, OCT-derived fractional flow reserve (FFR) might be superior to IVUS-derived FFR or angiography-derived FFR. Although the clinical implication of these approaches still needs further validation, they may have the potential to enable a comprehensive assessment of the characteristics of a lesion and its hemodynamic severity using a single imaging technique (68–70).

#### Bioresorbable Scaffold Implantation

In the treatment with bioresorbable scaffold (BRS), intravascular imaging played a major role in facilitating precise implantation of the device, which has thick and wide struts. OCT would be clinically more applicable for the optimized implantation than IVUS because the polymeric struts of BRS are scarcely visible by IVUS. Although the novel 60-MHz IVUS acquires higher-resolution (axial resolution <40 μm) images than conventional IVUS (71), it is still inferior to OCT in terms of visualization of BRS struts (72, 73). In PCI with BRS, the importance of an optimized implantation technique, the so-called PSP (prepare the lesion, sizing appropriately, and post-dilation), has been suggested to avoid clinical adverse events (74–76). Intravascular imaging is mandatory to fulfill these PSP criteria during PCI (77). OCT can provide a clear visualization of BRS struts, but it is unable to assess all vessel layers due to its low penetration depth. IVUS allows the visualization of the entire vessel wall enabling the calculation of its eccentricity and symmetry indices and the evaluation of vessel remodeling that subsequently occurs after BRS implantation. Serruys et al. demonstrated that BRS implantation was associated with a higher incidence of expansive remodeling and late lumen enlargement at 3 years compared to metallic stents in the ABSORB II trial (51). Although this favorable effect was not consistent in other ABSORB trials (78), it will be important to assess remodeling patterns at long-term follow-up after the full absorption of the deployed scaffolds.

#### Coregistration of IVUS and OCT

Several studies attempted to fuse IVUS and OCT images obtained by two different imaging catheters in a serial fashion and showed the synergistically high potential of multimodality imaging for the evaluation of plaque composition, but they also suggested the limitation to acquire strict coregistration images by separate two pullbacks (79, 80). To fuse IVUS and OCT images with two separate acquisition, it is necessary to detect several landmarks such as side branches and calcifications (81). Thereafter, it also takes time and effort to combine the two imaging modalities into a single image, a fact that makes impossible the broad application of this approach in research. A hybrid IVUS–OCT catheter system is warranted to enable accurate online coregistration and fusion of high-quality images obtained by the two imaging probes during a single pull-back.

## Recent Advances of IVUS and OCT, and Development of the Hybrid Catheter

### Development of a Hybrid IVUS–OCT Catheter

**Figure 3:**
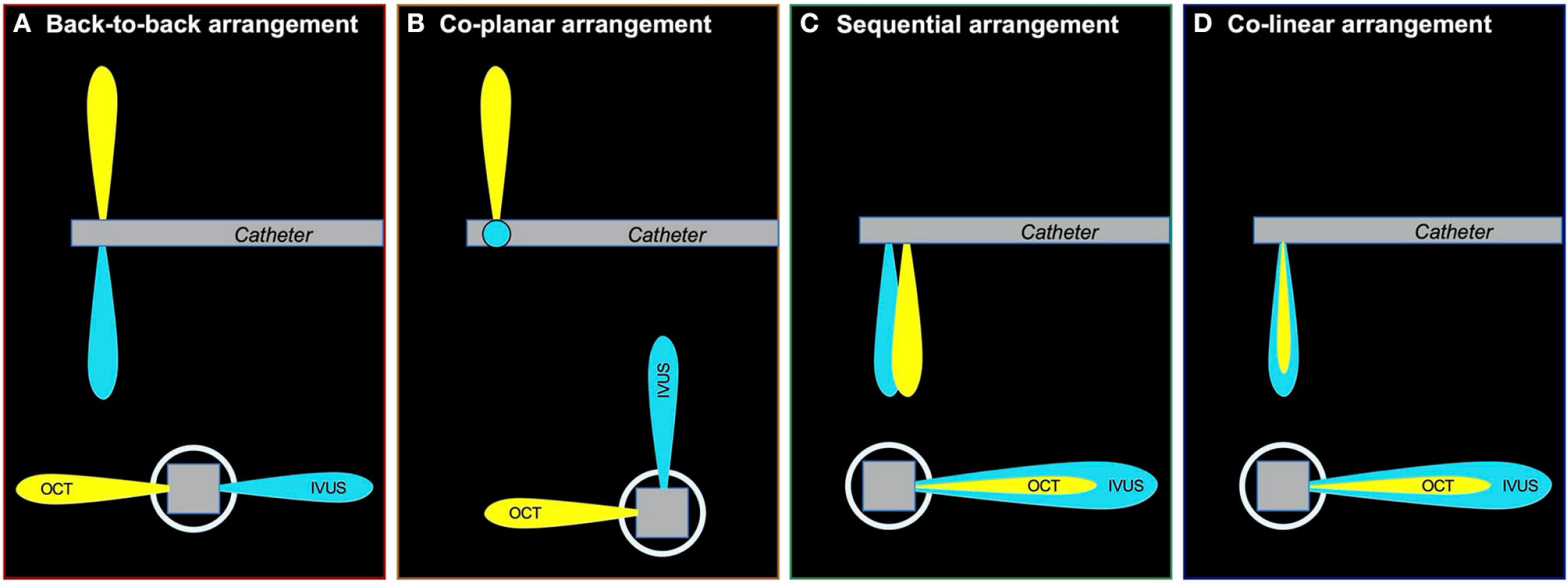
Various types of arrangements of IVUS and OCT transducer. There were three types of arrangements of IVUS and OCT transducer; **(A)**: back-to-back arrangement; **(B)**: coplanar arrangement; **(C)**: sequential arrangement; **(D)**: colinear arrangement. The colinear arrangement can acquire the strictest coregistration image of IVUS and OCT in three types of arrangements. IVUS, intravascular ultrasound; OCT, optical coherence tomography.

The first combined IVUS–OCT catheter was designed by Li et al. (28) and Yin et al. (30) and tested in a healthy rabbit aorta. However, the prototype had limitations that did not allow its use in clinical practice; the catheter was too large with a maximum outer diameter of 2.4 mm (7.2 Fr.); it did not allow the accurate coregistration of the OCT and IVUS images, and the generated IVUS images had increased noise due to electromagnetic interference by the motor (29, 30). In 2011, Yin et al. reported a modified combined miniaturized OCT–IVUS probe (82). The outer diameter was reduced to 0.69 mm, which fit in a 3.6 Fr. (1.18 mm) catheter sheath; this was achieved by arranging the position of the OCT probe and the IVUS transducer to longitudinal offset (sequential arrangement: Figure 3C). They tested the IVUS–OCT system *in vitro* in a human coronary artery specimen and *in vivo* in a rabbit abdominal aorta and a swine coronary artery (83).

Li et al. introduced another hybrid IVUS–OCT system in 2012 (84). They developed a coplanar IVUS–OCT hybrid catheter with a maximum outer diameter of 1.33 mm (4 Fr.) including the catheter sheath, allowing a more accurate simultaneous coregistration of IVUS and OCT compared to the sequential IVUS–OCT system (Figure 3B), even in the presence of cardiac motion. Validation of the prototype using human coronary arteries demonstrated improved good tissue characterization and plaque feature identification using histology as the gold standard. A variant IVUS–OCT probe with a shorter length of the rigid part of the catheter tip of 1.5 mm was shown by Li et al. and Ma et al. in 2013 (85, 86). That model had a back-to-back arrangement (Figure 3A) and allowed image acquisition at a higher frame rate of 20 fps compared to past models. In 2015, Li et al. showed an advanced IVUS–OCT prototype that acquired IVUS–OCT images with a frame rate of 72 fps, enabling assessment of a 72-mm coronary artery segment in 4 s; the prototype was validated *in vivo* in the aortas of atherosclerotic rabbits and *ex vivo* in cadaveric human coronary arteries (87). In 2018, the first clinical use of the hybrid IVUS–OCT catheter was reported by Sheth et al., showing beautiful coregistered images with clinically acceptable specifications with respect to the size, speed, and resolution.

### CONAVI: The Novasight Hybrid System

**Figure 4:**
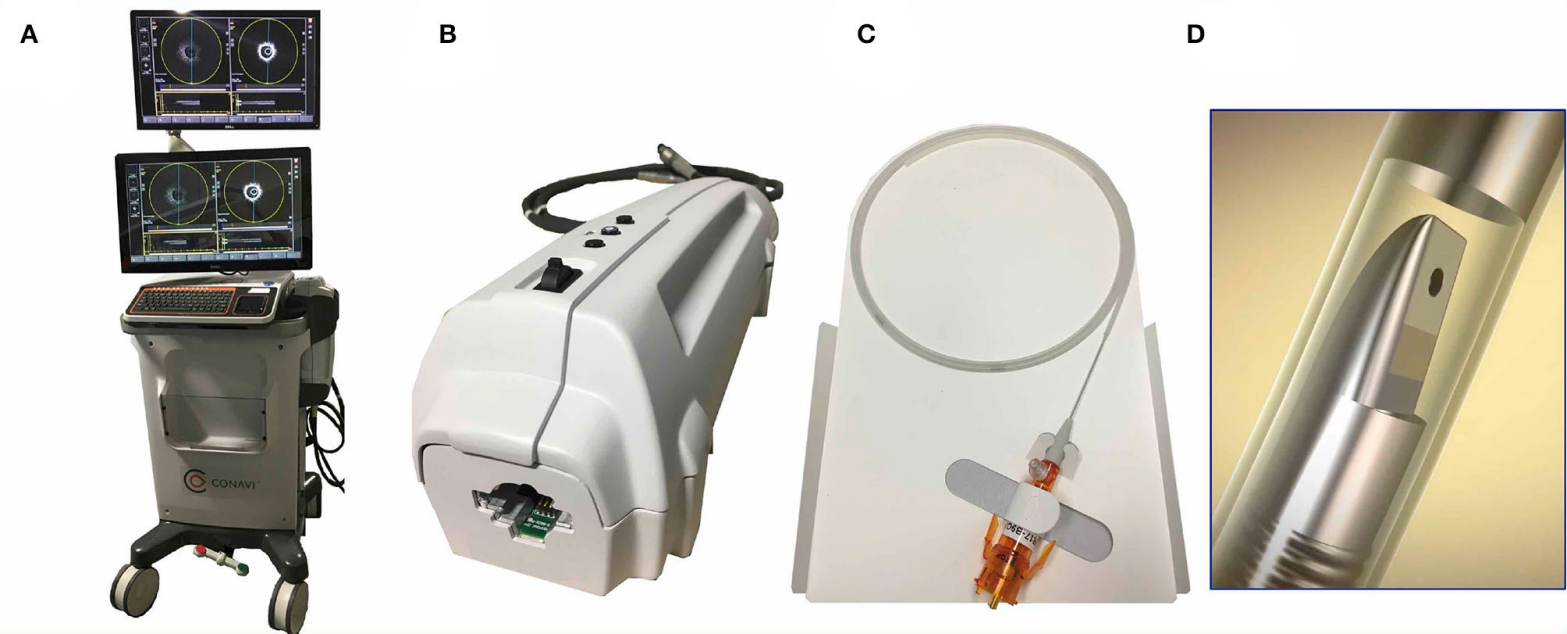
CONAVI Novasight hybrid imaging catheter; external appearances. **(A)** A whole appearance of system body. **(B)** Interface module. **(C)** Catheter technical specifications. **(D)** The transducer. IVUS, intravascular ultrasound; OCT, optical coherence tomography.

**Figure 5:**
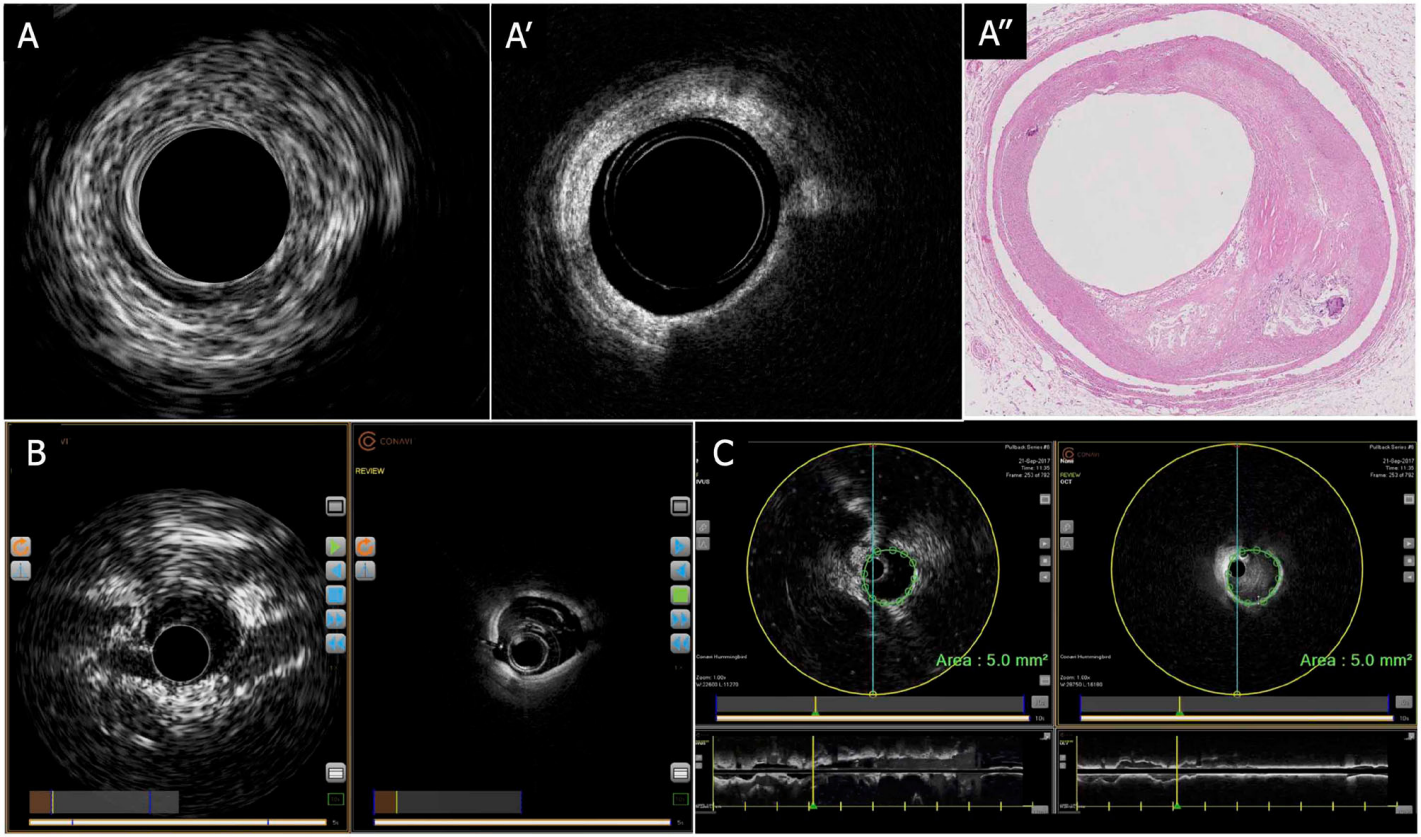
CONAVI Novasight hybrid imaging catheter; sample images. **(A)** Coregistered intracoronary images of superficial atheroma in IVUS (A-1) and OCT (A-2), and corresponding histopathology (A-3). **(B)** Preclinical *in vivo* images. **(C)** Measurements of lumen size by the coregistered image of IVUS and OCT. By default, any measurements (areas, distances) made in an IVUS image are automatically copied over into the OCT image and vice versa. The copied measurement can be removed. IVUS, intravascular ultrasound; OCT, optical coherence tomography.

The Novasight Hybrid™ System was developed by Conavi Medical Inc. (Toronto, Canada) and researchers at the University of Toronto (Figure 4). The product specifications are shown in Table 1. The catheter has a 1.7 Fr. tip and a 2.8 Fr. imaging window distally (3.3 Fr. catheter shaft for the proximal and middle sections of the catheter), and is compatible with a 0.014-inch guidewire. Colinear imaging design with overlapping IVUS and OCT was adopted (Figure 3D), which allows the user to visualize the vessel wall with both modalities at the same time and to inherently acquire accurately coregistered images (Figures 5A,B). Lumen or vessel size can be measured based on both IVUS and OCT cross-sectional images (Figure 5C). Pullback speed can be selected from 0 (manual control), 0.5, 1.0, 5, 10, and 25 mm/s with a maximum pullback length of 100 mm. In case of standalone IVUS imaging, the frame rate is 30 or 100 fps. In case of combined IVUS and OCT imaging, the pullback will be performed with a frame speed of 100 fps after blood clearance. The system does not have a setting that allows for the acquisition of OCT only, as there is little, if any, anticipated disadvantage to collecting IVUS at the same time. The maximum field of view radius is 6 mm derived from IVUS.

**Table 1:**
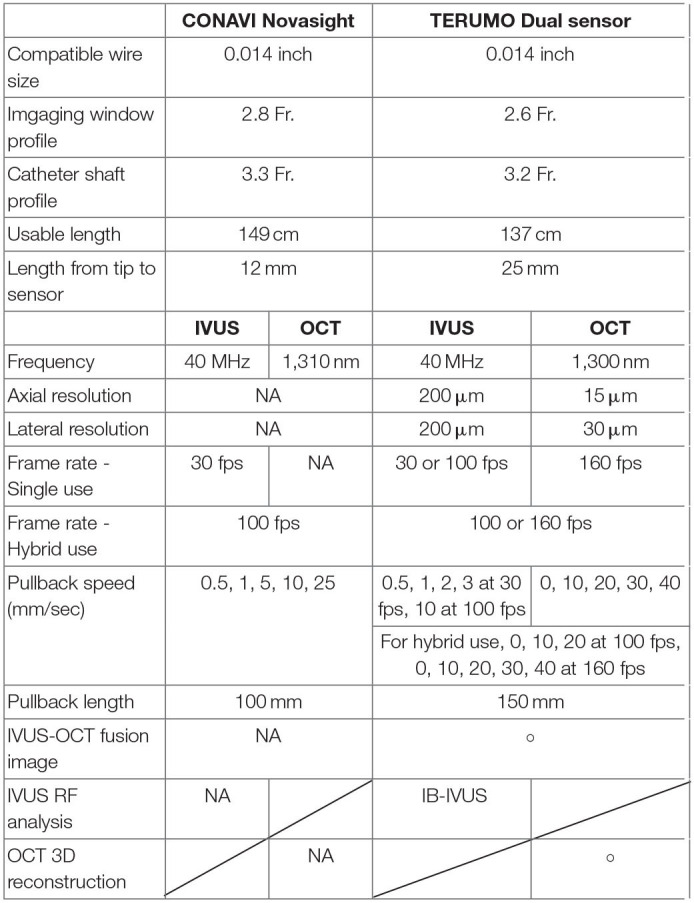
The specifications of CONAVI Novasight Hybrid and TERUMO Dual Sensor system.

*IVUS, intravascular ultrasound; OCT, optical coherence tomography; RF, radiofrequency backscatter; IB-IVUS, integrated-backscatter IVUS; 3D, three dimensional; NA, not applicable*.

The first clinical usage was reported by Sheth et al. in 2018 (88). In that case, the Novasight system could provide coregistered and co-aligned IVUS and OCT images in a patient with recent ST-segment elevation MI (STEMI) followed by PCI for a non-culprit lesion of the LAD. Lipid-rich plaques, bifurcations, and deeply embedded tissues were more clearly identified by IVUS images than OCT, whereas calcifications, stent struts, and fine dissections were more clearly identified by OCT imaging.

The Novasight system is currently FDA 510(k) cleared and has Health Canada approval. A prospective observational study using the Novasight hybrid imaging catheter has been completed and demonstrated its feasibility and efficacy for diagnostic purposes and PCI guidance in 20 patients with a chronic coronary syndrome or ACS (NCT03484975).

### TERUMO: The Dual Sensor

**Figure 6:**
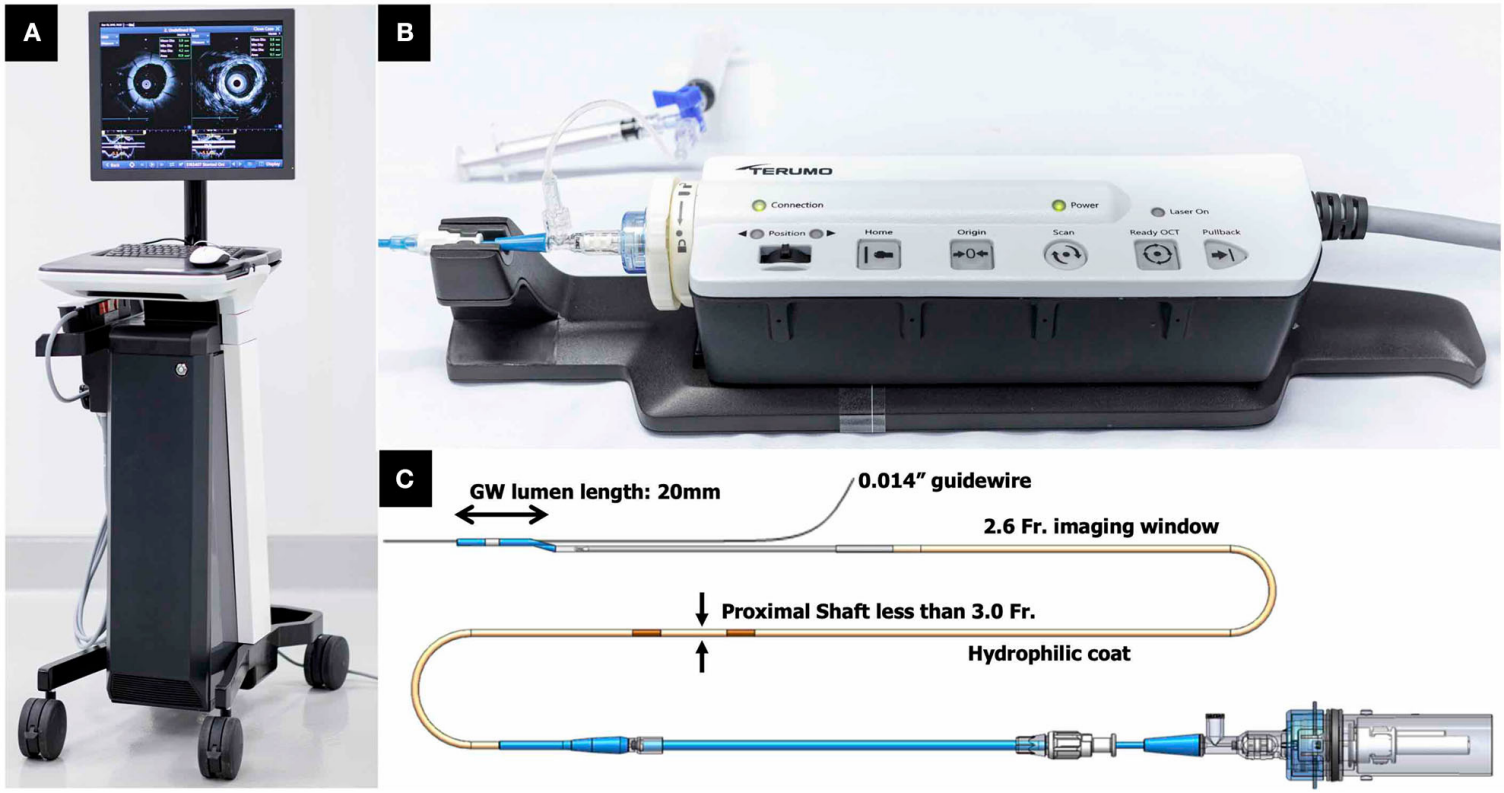
TERUMO hybrid IVUS–OCT catheter system; external appearances. **(A)** A whole appearance of system body. **(B)** Interface module. **(C)** Catheter technical specifications. IVUS, intravascular ultrasound; OCT, optical coherence tomography.

**Figure 7:**
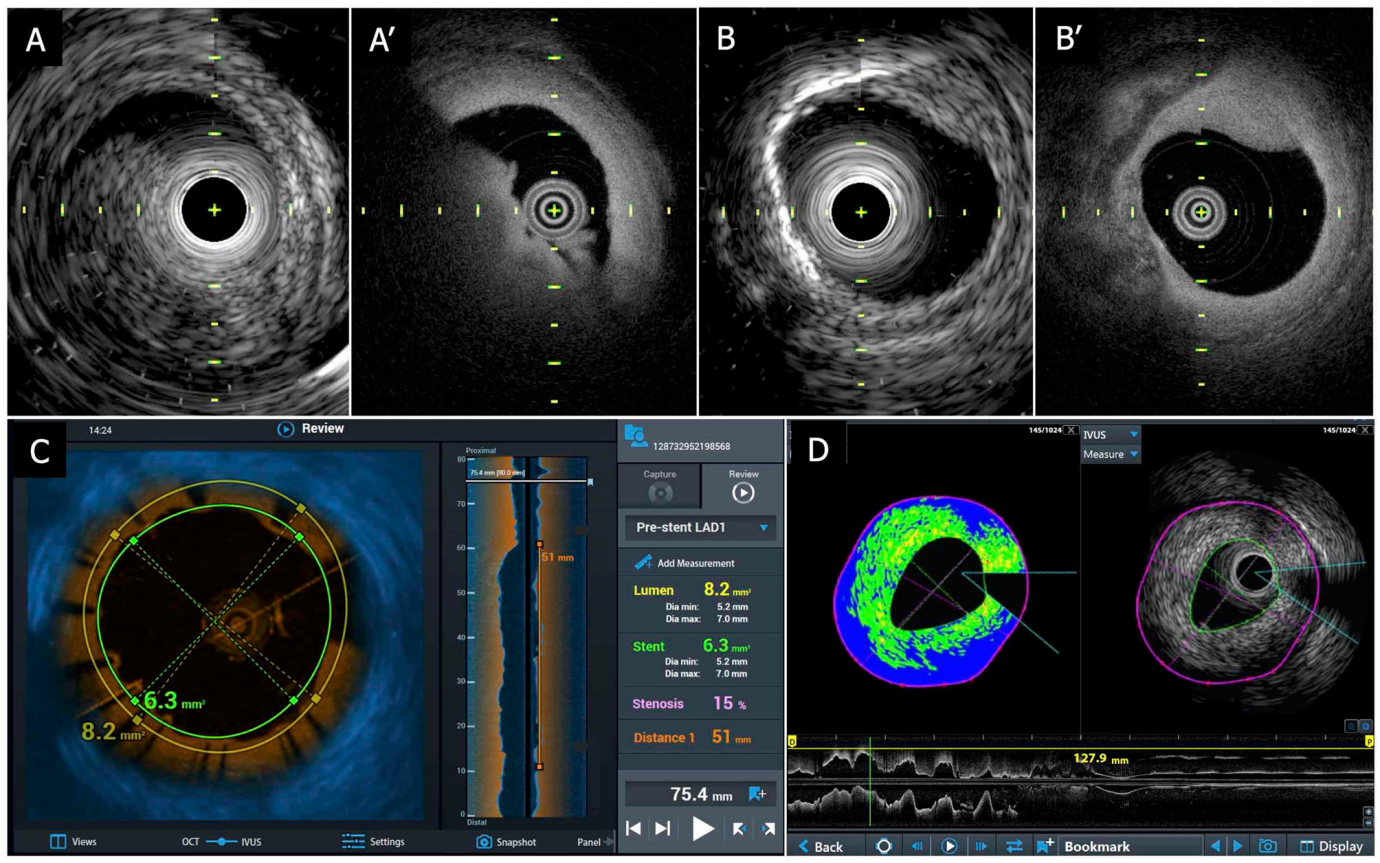
TERUMO hybrid IVUS–OCT catheter system; sample images. **(A)** Coregistered intracoronary imaging of thrombus in IVUS (A-1) and OCT (A-2) in a cadaver coronary artery. **(B)** Coregistered intracoronary imaging of calcification in IVUS (B-1) and OCT (B-2) in a cadaver coronary artery. **(C)** Fusion image of IVUS and OCT. **(D)** IB-IVUS image with OCT. IVUS, intravascular ultrasound; OCT, optical coherence tomography; IB, integrated backscatter.

The Dual Sensor hybrid IVUS–OCT catheter system is developed by TERUMO (Tokyo, Japan) by merging IVUS and optical frequency domain imaging (OFDI) probes, which are already clinically available and incorporated in the AltaView (PMDA approval) and FastView (PMDA and CE mark approval), respectively. A sequential arrangement of an IVUS transducer and optical lens with the distance of ~0.3 mm is adopted (Figure 3C). The catheter is compatible with a 0.014 guidewire and has a diameter 2.6 Fr. (3.0 Fr. catheter shaft) (Figure 6). The preliminary product specifications are shown in Table 1. The IVUS probe has an axial resolution of 120 μm, while the OCT has an axial resolution of 20 μm (Figures 7A,B). The high frame rate of 100 or 160 fps enables a pullback speed of up to 40 mm/s (0, 10, 20, 30, and 40 mm/s) and studied segments with a length of 150 mm on IVUS and OCT. The image acquisition method of the simultaneous coregistration of IVUS and OCT is the same as that of OCT. The acquired images will be shown not only side by side but also in one fusional image of IVUS and OCT (Figure 7C). Integrated-backscatter IVUS (IB-IVUS) analysis will also be available, which will offer further information about tissue and plaque characteristics (Figure 7D). Online OFDI 3D reconstruction will facilitate comprehensive evaluation of complex coronary artery structures.

The device has been tested in postmortem coronary arteries, and its output was compared to contemporary IVUS as well as OCT (Figure 7). In this hybrid catheter, IVUS or OCT can be separately used. Operators can select those functions according to circumstances during the procedure. For instance, in a case of left main coronary artery disease, the operator can use the IVUS function for the purpose of assessing the severity and guide treatment (25), whereas OCT can be utilized to assess the final results and detect and treat underexpansion/malapposition and/or carina shift in the bifurcation (57, 89, 90). The cost is expected to be similar to that of OFDI.

## Potential Clinical Application of a Hybrid IVUS–OCT Catheter

### For Detecting “High-Risk Plaques”

Acute coronary syndrome (ACS) remains one of the leading causes of death even in the contemporary era (91). It's well-known that, in most cases, myocardial infarction can be caused not by the gradual progression of stenosis, but by a sudden thrombotic occlusion (92). Virmani et al. reported three major distinct processes of ACS in histopathological studies, which are plaque rupture, erosion of intima, and calcified nodule (93, 94). Among those, plaque rupture occurring in lesions with a TCFA phenotype is the most frequent cause of ACS and is responsible for 60–70% of all ACS (94). Intensive medical therapies (e.g., aggressive LDL cholesterol lowering) and/or intensive lifestyle changes can improve the outcomes of the patients with high-risk plaques (95–98).

TCFA is recognized as a plaque with a necrotic core and an overlying thin fibrous cap with thickness of <65 μm that contains macrophages and smooth muscle cells (99). Narula et al. reported in a postmortem study that plaques with fibrous caps thicker than 84 μm are stable plaques, and that in the majority of the cases, the cap thickness in TCFA ranged between 54 and 84 μm (100). In any case, the evaluation of TCFA would be technically challenging by grayscale IVUS because of its limited spatial resolution of 100–150 μm, which exceeds the thickness of TCFA (101). Radiofrequency analysis of IVUS (RF-IVUS, e.g., virtual histology IVUS [VH-IVUS] by Philips Healthcare) appears more accurate than grayscale IVUS in detecting plaque components. A classification scheme has been proposed to define plaque phenotypes and detect TCFA using the RF-IVUS estimations and has been extensively used in prospective studies of coronary atherosclerosis (102). Similar to the previously mentioned PROSPECT (18) and ATHEROREMO-IVUS trials (19), the VIVA (VH-IVUS in Vulnerable Atherosclerosis) study also showed that VH-IVUS TCFA was significantly associated with the increased risk of MACE (103).

On the other hand, OCT has the highest spatial resolution (axial resolution 15–20 μm) among intracoronary imaging devices, which enables the precise measurement of the cap thickness (104–106). OCT has been well-validated, and its estimations correlated well with the corresponding pathophysiological findings (107, 108). Furthermore, some studies reported that the OCT-detected TCFA was also clinically associated with the future occurrence of MACE (26, 109, 110). However, OCT also has limitations as it has poor tissue penetration. In addition, the OCT signal is attenuated by lipid tissue and macrophages (111). Therefore, OCT can often misclassify fibrotic rich plaques with increased superficial macrophage accumulations as TCFA. In addition, OCT cannot assess the plaque burden in lipid-rich plaques, which is a strong predictor of worse outcomes (18, 19, 103).

The quantitative analysis of plaque burden would be well-assessed by IVUS, especially by IVUS-RF analysis. Recently, near-infrared spectroscopy intravascular ultrasound (NIRS-IVUS) was introduced to more accurately detect lipid core plaques (112–114).

Several previous studies reported the great benefit of the combined use of OCT and VH-IVUS for detecting TCFA compared to the single use of either OCT or VH-IVUS (107, 111, 115). Nakano et al. showed that the combined use of IVUS-RF and OCT gave the highest performance to detect TCFA (area under the curve [AUC] 0.93, 95% CI 0.85–1.00) compared with standalone OCT (AUC 0.88, 95% CI 0.76–0.99) or RF-IVUS (AUC 0.82, 95% CI 0.68–0.95) in the *ex vivo* autopsy study (116). Futures studies are expected to examine the efficacy of the combined IVUS–OCT imaging catheters in detecting vulnerable plaques.

In addition, wall shear stress would play an important role in both a plaque progression and a plaque rupture (117–119). In the assessment of wall shear stress, the fusion of intracoronary imaging data and coronary angiography or computed tomography coronary angiography (CTCA) is needed to make a high-resolution coronary artery model (120–122). The consensus paper on the assessment of wall shear stress stated that studies aiming to investigate the effect of wall shear stress on plaque progression and changes in plaque composition should preferably create coronary artery models using the fusion of IVUS/NIRS-IVUS and coronary angiography or CTCA (123, 124). On the other hand, studies aiming to investigate the influence of the local wall shear stress on plaque microcharacteristics should preferably create the 3D lumen using the fusion of OCT and biplane angiography or CTCA (125). The hybrid IVUS–OCT catheter also could allow one to make a more accurate coronary artery model and may provide new insight into the association between wall shear stress and a plaque progression/regression.

### Imaging During PCI and Stent Optimization

Although the routine use of intracoronary imaging is not currently recommended to guide PCI in simple lesions, it appears to be useful for optimizing PCI results in several clinical settings. The consensus document from the EAPCI recommended the use of intracoronary imaging in the setting of ACS, left main coronary artery (LMCA) disease, two stents bifurcation, implantation of bioresorbable scaffold, and patients with renal dysfunction (for IVUS) (45, 46). In the current ESC/EACTS guidelines of myocardial revascularization, the use of IVUS or OCT to optimize stent implantation is recommended in selected patients as Class IIa indication (25).

Hybrid IVUS–OCT catheters have a high potential to support procedures in a wide range of circumstances by its high diagnostic efficacy derived from the combination of the two imaging modalities. Therefore, the hybrid catheter is expected to be widely utilized in daily clinical practice. In the following section, we describe the potential value of this hybrid catheter in guiding PCI in the most challenging lesions such as LMCA disease and multivessel coronary artery disease (MVCAD).

### Hybrid IVUS–OCT Catheter for Left Main Coronary Artery Disease

LMCA disease can involve either the ostium or the main vessel or its bifurcation; the left main stem supplies with blood most of the myocardium, and thus, treatment failure can lead to critical peri-procedural complications during PCI (126–129). In the EXCEL trial, PCI was non-inferior to CABG in terms of the primary endpoint (all-cause death, stroke, or MI) at 3 years (HR: 1.00, 95% CI 0.79–1.26) (128) and at 5 years (HR: 1.19, 95% CI 0.95–1.50) (130) in patients with LMCA disease and low or intermediate SYNTAX scores. Of note, the rate of ischemia-driven revascularization was significantly higher in the PCI arm than in the CABG arm at 5 years (16.9% in the PCI arm and 10.0% in the CABG arm, HR: 1.84, 95% CI 1.39–2.44). Although IVUS guidance was strongly recommended in this trial, it was used only in 77.2% of the cases in the PCI arm.

Intravascular imaging is useful in assessing the stenosis severity and guiding PCI in patients with LMCA disease. As a threshold for revascularization, a minimum lumen area (MLA) of <6.0 mm^2^ by IVUS has been recommended (131), which nearly corresponded to an FFR of <0.80 in Western countries (25, 132), whereas in the Asian population, the threshold of MLA is relatively smaller (4.5–4.8 mm^2^) compared to that of the Western population (133, 134). The sub-analysis of the MAIN-COMPARE (Revascularization for Unprotected Left Main Coronary Artery Stenosis: Comparison of Percutaneous Coronary Angioplasty vs. Surgical Revascularization) registry clarified the favorable outcomes of IVUS-guided PCI compared to angiography-guided PCI in patients with unprotected LMCA disease (126). Hernandez et al. also demonstrated that the use of IVUS significantly reduced MACE compared to those of PCI without IVUS guidance in a registry study (127).

On the other hand, the value of OCT in LMCA disease is still unclear. The ESC/EACTS guideline of myocardial revascularization recommended only IVUS for the assessment of the severity and treatment optimization in LMCA disease (class IIa indication), whereas OCT is not mentioned regarding LMCA disease (25). OCT-guided PCI seems to be less feasible for LMCA disease than IVUS because of the large vessel diameter and the limited penetration depth of OCT. Moreover, blood clearance can be sometimes difficult especially in proximal LMCA disease. Nevertheless, it has to be acknowledged that OCT usage may have some advantages. OCT in LMCA bifurcation PCI can assess acute incomplete stent apposition (ISA) after stent implantation followed by the kissing-balloon technique (KBT) (57, 135). In order to avoid the so-called “metal carina,” the re-crossing wire following stent implantation should pass through the most distal cell of the stent on the jailed side-branch ostium in most cases. OCT, especially 3D reconstruction, can show stent struts in detail with a high resolution (89). In the OPTIMUM trial, Onuma et al. reported that 3D OCT-guided PCI and re-crossing through the optimal strut cell after stent implantation followed by KBT significantly reduced acute ISA at the bifurcation compared to angiography-guided PCI (19.5 vs. 27.5%, *p* = 0.008) in patients with bifurcation lesion including LMCA disease (preliminary data) (136, 137). In addition, final stent optimization excluded the possibility of stent distortion, or stent underexpansion can be assessed easier in OCT that has a much higher resolution than IVUS. Furthermore, OCT-derived FFR can be utilized in the future to evaluate lesion severity and the residual ischemic risk after the procedure (69, 70).

By using hybrid IVUS–OCT systems, we can use each function according to the process comprehensively. For example, OCT will be utilized for re-crossing wire, the final stent optimization, and/or physiologically assessments, while IVUS will be utilized for the indication, the stent/balloon sizing, and/or deciding the location of the stent landing zone.

#### Hybrid IVUS–OCT Catheter for Multivessel Coronary Artery Disease

With regards to multivessel PCI, IVUS-guided PCI is recommended as Class IIa indication in the latest ESC/EACTS guideline of myocardial revascularization (25). In the SYNTAX II trial, recent technical and procedural developments significantly improved clinical outcomes in terms of 1-year major adverse cardiac and cerebrovascular events [MACCE (composite of all-cause death, cerebrovascular event, any MI, and any revascularization)] compared to those of the PCI arm of the original SYNTAX-I trial (HR: 0.58, 95% CI 0.39–0.85), and those outcomes were equivalent to those of the CABG arm of the original SYNTAX-I trial (HR: 0.91, 95% CI 0.59–1.14), in patients undergoing multivessel PCI (138, 139). The difference in the results between SYNTAX-I and -II trials should be attributed to the differences in the treatment strategies between the SYNTAX II trial and the SYNTAX-I trial. In summary, there are six differences between the two studies: patient selection based on the SYNTAX II score, physiological assessment of stenotic lesion, use of intracoronary imaging for complex procedures, PCI of chronic total occlusion performed by an expert, use of current-generation DES, and optimal medical treatment before/after PCI (140). In other words, PCI could become equivalent to CABG only in cases fulfilling these criteria in the field of MVCAD, and the use of an intracoronary imaging is mandatory in this setting. MVCAD often shows a mixture of various types of lesions (e.g., with bifurcation lesion, severe calcification, and/or lipid-rich plaques); therefore, different strategies might be required according to each lesion characteristic. The hybrid IVUS–OCT will be able to assist operators to tailor treatment strategy according to lesion types. In addition, intracoronary imaging-derived FFR may be used to assess lesion severity and identify those that need treatment, and estimate the residual ischemic risk after stent implantation, which is also one component of “best practice PCI” for MVCAD.

## Limitation

Despite the fact that many interventional cardiologists are aware of the potential prognostic and clinical value of intravascular imaging-guided PCI, the application of intravascular imaging is still low in recent clinical practice especially in Western countries (141). According to a web-based survey (141), the most common reason for the underuse of intravascular imaging is its high cost (65.9%), followed by the prolongation of the diagnostic procedure or intervention (35.0%), albeit risk of complications (9.5%) and absence of established clinical value (8.3%) were, overall, the least commonly reported limiting factors. However, it was also reported that IVUS-guided PCI could contribute to the favorable incremental cost-effectiveness compared to angiography-guided PCI in the dedicated economic analysis (142).

The hybrid catheter has a marginal incremental cost to build relative to a single modality imaging catheter that would not greatly impact the overall cost-effectiveness of intravascular imaging. The consoles and patient interface modules have costs that are associated with supporting ultrasound electronics as well as the optical components for OCT, and thus have a more noticeable, yet still modest, incremental cost over single modality systems to provide this advanced dual modality imaging capability.

For the purpose of detecting a high-risk plaque, a recent LRP trial demonstrated the impact of a Lipid Core Burden Index derived from NIRS-IVUS on future cardiac events, which was independent of intravascular ultrasound plaque burden or minimum lumen area (113). Further clinical trials will be needed to compare the hybrid IVUS-OCT system vs. NIRS-IVUS in terms of the prognostic performance in patients at risk.

## Conclusion

Although the routine use of intravascular imaging is not currently recommended (143), its use is expected to reduce events in complex PCI. Moreover, in the era of emerging novel pharmacotherapies, a meticulous evaluation of plaque morphology would be required to discriminate the potential population in need (144). The hybrid IVUS–OCT systems have the potential to assess plaque morphology and PCI results. “IVUS or OCT, which should be used?”—this question has been frequently repeated up to now (6, 145). The hybrid IVUS–OCT system could be the answer in the near future.

## Author Contributions

MO gathered, reviewed, and interpreted available literature, wrote the first draft of the article, and contributed to all revisions of the manuscript. PS and YO organized the project, gathered and interpreted the literature, and contributed to all revisions of the manuscript. HK, HH, CG, RW, NK, KT, PC, RM, and MT gathered and reviewed the literature and contributed to all revisions of the article. JP, JW, WW, FS, and CB gathered and interpreted the literature and contributed to all revisions of the manuscript as experts of the field. IM and BC gathered and interpreted the data and contributed to revisions of the manuscript. All authors provided final approval of the version to be published.

## Conflict of Interest

IM was employed by the company Terumo Corporation, Tokyo, Japan. BC has the following conflicts of interest with Conavi Medical Inc.: co-founder, employee, significant equity ownership, patent/royalty rights, and research funding. PS reports personal fees from Biosensors, Micel Technologies, Sinomedical Sciences Technology, Philips/Volcano, Xeltis, and HeartFlow, outside the submitted work. JP reports personal fees and non-financial support from Philips/Volcano, outside the submitted work. The remaining authors declare that the research was conducted in the absence of any commercial or financial relationships that could be construed as a potential conflict of interest.

## Abbreviations

3D: Three-dimensional
ACS: acute coronary syndrome
BRS: bioresorbable scaffold
BMS: bare-metal stent
CTCA: computed tomography coronary angiography
DES: drug-eluting stent
EAPCI: European Association of Percutaneous Cardiovascular Interventions
EEM: external elastic membrane
ESC: European Society of Cardiology
FFR: fractional flow reserve
IB: integrated backscatter
IVUS: intravascular ultrasound
KBT: kissing-balloon technique
LAD: left anterior descending artery
LMCA: left main coronary artery
LMWD: low-molecular-weight dextran
MACE: major adverse cardiac events
MACCE: major adverse cardiac and cerebrovascular events
MI: myocardial infarction
MVCAD: multivessel coronary artery disease
NIRS: near-infrared spectroscopy
OCT: optical coherence tomography
OFDI: optical frequency domain imaging
PCI: percutaneous coronary intervention
RF: radiofrequency data
TCFA: thin-cap fibroatheromas
TVF: target-vessel failure
VH: virtual histology.
