# Erratum: Advances in IVUS/OCT and Future Clinical Perspective of Novel Hybrid Catheter System in Coronary Imaging

**DOI:** 10.3389/fcvm.2020.594899

**Published:** 2020-09-16

**Authors:** Frontiers Production Office

**Affiliations:** Frontiers Media SA, Lausanne, Switzerland

**Keywords:** intravascular ultrasound, optical coherence tomography, hybrid IVUS–OCT catheter, intracoronary imaging, vulnerable plaque, percutaneous coronary intervention

Due to a production error, there was a mistake in Table 1 as published. The table was incorrectly formatted. The corrected Table 1 appears below.

**Table 1:**
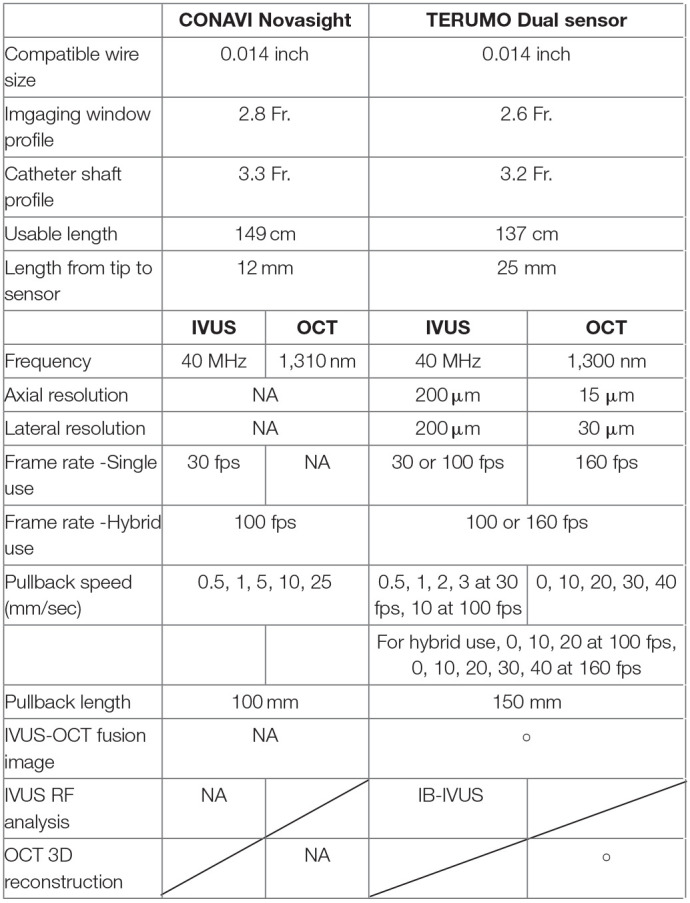
The specifications of CONAVI Novasight Hybrid and TERUMO Dual Sensor system.

*IVUS, intravascular ultrasound; OCT, optical coherence tomography; RF, radiofrequency backscatter; IB-IVUS, integrated-backscatter IVUS; 3D, three dimensional; NA, not applicable*.

The publisher apologizes for this mistake. The original article has been updated.

